# CXCL13 as a Biomarker of Complex Common Variable Immunodeficiency

**DOI:** 10.1007/s10875-025-01963-2

**Published:** 2025-11-25

**Authors:** Ioasaf Karafotias, Helene Martini, Charlotte V. Lee, Terrence T. J. Hunter, Padmalal Gurugama, Mary Guckian, Rachael Steven, Stephen Jolles, Mark Peakman, David Fear, Mohammad A. A. Ibrahim

**Affiliations:** 1 Department of Immunological Medicine, King’s College London, King’s Health Partners, King’s College Hospital NHS Foundation Trust, School of Immunology and Microbial Sciences, Denmark Hill, London, SE5 9RS UK; 2 Synnovis (formerly Viapath), King’s College Hospital, Denmark Hill, London, SE5 9RS UK; 3 Immunodeficiency Centre for Wales, University Hospital of Wales, Cardiff, UK; 4 Peter Gorer Department of Immunobiology, School of Immunology & Microbial Sciences, King’s College London, London, UK

**Keywords:** Common variable immunodeficiency (CVID), C-X-C Motif Chemokine Ligand 13 (CXCL13), T follicular helper cells (Tfh), Biomarker, Immunodeficiency

## Abstract

**Background:**

Common Variable Immunodeficiency (CVID) is a group of heterogeneous disorders with common denominators of impaired antibody production and function, and recurrent infections. Currently, prognostic biomarkers for CVID are limited. CXCL13 is a critical regulator of germinal centre responses and antibody production, with T follicular helper (Tfh) cells as a major source, and acts as a potent B cell chemoattractant. Serum levels of CXCL13 are increased in chronic inflammatory conditions and malignancy.

**Objectives:**

We aimed to explore whether serum CXCL13 levels are altered in CVID and whether they can categorise the patients based on their clinical and immune phenotype.

**Methods:**

We compared the serum levels of CXCL13 between CVID and healthy donors (HD) and associated them with the clinical and immune phenotype of the patients.

**Results:**

The serum levels of CXCL13 were higher in CVID, especially in female patients, as compared to HD, and were positively correlated with the number of clinical complications in CVID and the total peripheral circulating Tfh cells (cTfh). CVID patients with higher levels of CXCL13 were more likely to have clinical complications and/or high frequency of CD21^low^ B cells or low frequency of switched memory B cells.

**Conclusions:**

CXCL13 can categorise heterogeneous patients with CVID and be used as a biomarker of complex disease.

**Supplementary Information:**

The online version contains supplementary material available at 10.1007/s10875-025-01963-2.

## Introduction

 Common Variable Immunodeficiency (CVID) is a heterogeneous group of primary immunodeficiency disorders, having in common defective antibody production and function together with recurrent infections [1, 2]. Among patients with CVID, there are patients with recurrent infections only (approx. 2/3 of cases) and better prognosis compared to the patients with immune dysregulation and worse prognosis [3, 4]. Patients from the last group (complex CVID) may have one or more complications such as: autoimmunity (including autoimmune cytopenia), abnormal lymphoproliferation leading to persistent, benign lymphadenopathy, splenomegaly, or granulomata (including granulomatous interstitial lung disease – GLILD), as well as lymphoma, and/or enteropathy [5–7]. In addition, untreated/long-standing lower respiratory tract infections may lead to bronchiectasis [5]. Due to this heterogeneity, there have previously been several attempts to categorise CVID patients [6, 8–10]. One of these has been the EUROclass classification, where the high frequency of CD21^low^ B cells and low frequency of switched memory B cells are associated with a more complex clinical phenotype [8]. It has also been identified that CVID is associated with higher levels of various inflammatory markers, including CRP and β2-microglobulin [11–16]. The latter has also been identified to reflect increased disease burden [13].

During the humoral immune response, the chemokine CXCL13 directs B cells across a chemotactic gradient towards the germinal centres and is an important regulator of antibody formation [17]. It participates in the maturation of B cells, the generation of germinal centres in the secondary lymphoid organs and directs the B cells towards the light zone of the germinal centre [18–22]. During chronic inflammation, CXCL13 is essential for the formation of ectopic lymphoid follicles and tertiary lymphoid organs in non-lymphoid tissues [22–25].

CXCL13 has also been found to be elevated in several acute or chronic inflammatory conditions and malignancies such as HIV, HCV, candidiasis, rheumatoid arthritis, systemic lupus erythematosus, Sjögren’s syndrome, prostatic or breast cancer and in Non-Hodgkin B cell lymphoma [25–32]

T follicular helper cells (Tfh) are known to be a major source of CXCL13 in the lymphoid follicles, expressing CXCR5, the receptor for CXCL13 [33–35]. CXCL13 and Tfh cells cooperate in the germinal centre during the process of antibody formation [33–35]. Based on the transcription factors they express and the cytokines they secrete, Tfh cells have been subdivided into 3 major subsets (Tfh1, Tfh2 and Tfh17). Tfh2 and Tfh17 induce the production of immunoglobulins by naïve B cells and regulate isotype switching, hence are termed “efficient” helpers, while Tfh1 do not, and are termed “inefficient” helpers for antibody formation [36, 37]. All 3 major subsets are further subdivided based on their expression of the molecules ICOS and PD1, which indicate activated Tfh cell subsets contributing to antibody formation [38, 39]. Tfh cells have recently been found to be upregulated in CVID and have been used as a therapeutic target to try to suppress the chronic inflammation [40–42].

Based on the above, we hypothesised that the serum levels of CXCL13 might be altered in CVID and could help stratify the heterogeneous patients with this disease. To investigate this, we aimed to:

- compare the levels of CXCL13 in the serum of patients with CVID to those of healthy individuals.
- explore whether the CXCL13 serum levels can categorise the patients with CVID based on their clinical and immune phenotype.
- assess whether there is a correlation between CXCL13 and circulating Tfh (cTfh) cell subsets in patients with CVID.

## Materials and Methods

### Recruitment

The study protocol was approved by the Health Research Authority (HRA) and Health and Care Research Wales (HCRW) ethics committee (Approval REC reference: 20/YH/0015, IRAS 232824), complying with the Declaration of Helsinki. All participants signed and provided informed consent upon enrolment in the study. The patients with CVID were selected by fulfilling the diagnostic criteria for CVID, as derived from the 1999 ESID/PAGID diagnostic criteria [43].

Healthy donors (HD) – those with normal immunoglobulin levels and inflammatory markers, and no diagnosed illnesses – were selected randomly.

All participants were assessed clinically to exclude those with manifestations of acute infection. As an inflammatory marker, CRP levels in each were measured concurrently with the CXCL13 levels. There was no statistically significant age difference between the patients with CVID and the healthy participants in total and among the same gender (Table S1, supplementary material).

Venous blood samples were collected at convenience/random time points from patients with CVID (*n* = 76) and patients with X-linked Agammaglobulinemia (*n* = 7) visiting routinely, either the outpatient Primary Immunodeficiency Clinics of the Department of Clinical Immunology and Allergy, or the Programmed Investigation Unit immediately before receiving immunoglobulin replacement infusion at King’s College Hospital, London, UK [44]. The median time between repeated measurements was 5 months (Range: 0.5–32 months).

Blood samples from volunteers (*n* = 52) recruited via e-mailed advertisement and poster distributed across the Viapath (now Synnovis) diagnostic laboratories at King’s College Hospital were obtained and used as controls. The demographics of the participants are shown in Supplementary Table S1.

By consulting the electronic patient records, patients with CVID were assessed for the presence of any of the disease complications, i.e. bronchiectasis, granulomatous disease, autoimmune disease, cytopenia, splenomegaly, hepatopathy, interstitial lung disease, lymphadenopathy, and malignancy (Table 1) [6, 9, 45]. The patients, for whom confirmation for the presence or absence of a given complication was not possible (see “data” in Table 1 footnote), were excluded from the analysis of the possible association of CXCL13 or cTfh cell subsets with that complication. The collected blood samples were introduced immediately into pyrogen-free tubes and were allowed to clot at room temperature (RT) for 45 min before being centrifuged for 10 min at 3000 rpm and at RT.

**Table 1:** Clinical and Immunologic characteristics of the cohort of patients with CVID

	Data*/Total CVID patients	Case frequency (%)	Female case frequency (%)
Infections	**59†**/76	**12††**/59 (20)	**5**/12 (42)
Any complication	**67**/76	**55**/67 (82)	**31**/55 (56)
Splenomegaly	**72**/76	**34**/72 (47)	**15**/34 (44)
Lymphadenopathy	**63**/76	**20**/63 (32)	**12**/20 (60)
Granulomata	**66**/76	**16**/66 (24)	**12**/16 (75)
Interstitial Lung Disease (ILD)	**66**/76	**14**/66 (21)	**11**/14 (79)
Hepatopathy	**72**/76	**7**/72 (10)	**3**/7 (43)
Autoimmunity	**70**/76	**26**/70 (37)	**17**/26 (65)
Cytopenia	**74**/76	**20**/74 (27)	**16**/20 (80)
Enteropathy	**74**/76	**14**/74 (19)	**10**/14 (71)
Bronchiectasis	**67**/76	**27**/67 (40)	**15**/27 (56)
CD21^low^ (% of B cells)	**45/**76	**11**/45 (24)	**6**/11 (55)
smB- (% of B cells)	**45**/76	**17**/45 (38)	**9/17** (53)
IgG levels (prior to replacement)	**57**/76	**57**/76	**31**/57 (54)

* “Data” represents the number of patients having confirmed the presence or absence of the above parameters. All ILD cases were of the GLILD type and 5 hepatopathy cases were granulomatous liver disease. As per the criteria defined by the EUROclass classification “CD21low” represents the number of patients with high frequencies of CD21low B cells, and “smB-“ represents the number of patients with low frequencies of switched memory B cells

† all patients with verified infections; †† patients with infections only

### Enzyme-Linked Immunosorbent Assay (ELISA)

CXCL13 levels in the serum of the participants were measured by ELISA (Human CXCL13/BLC/BCA-1 Quantikine ELISA Kit by R&D) containing anti-human CXCL13 antibody, according to the manufacturer’s protocol [46]. The concentrations of CXCL13 were measured in pg/ml using EMax^®^ Plus Microplate Reader and SoftMax Pro 7 Software.

For the purposes of this research project, we calculated a reference range for the values of CXCL13 in HD. Since the values of CXCL13 did not exhibit a normal distribution, we log-transformed them and, based on the central limit theorem, calculated the reference range via the equation: reference range of CXCL13 (pg/ml) = mean +/- 2*SD, where SD stands for Standard Deviation of the mean CXCL13 value. The mean of the log-transformed CXCL13 values was 1.777 and the SD was 0.2444. Based on the equation, the upper limit of the normal reference range of serum CXCL13 was 184 pg/ml.

### CRP, IgG and β2-Microglobulin Measurements

The serum levels of CRP, IgG and β2-microglobulin – measured concurrently with the CXCL13 for each participant – were obtained via the Hospital’s Electronic Patient Records (EPR). The Viapath diagnostic Biochemistry and Immunology laboratories carried out all measurements. CRP in serum was quantitatively determined by immunoturbidimetric assay and normal CRP was considered any value of < 5 mg/l, while β2-microglobulin was measured on the Optilite platform and values of < 2.40 mg/l were considered normal [47, 48]. Serum levels of IgG between 6.34 and 18.11 g/l were considered within the normal reference range.

### Sample Collection, Staining and Flow Cytometry for Measurement of cTfh Cell Subset Frequencies by Flow Cytometry

Data on the frequencies of cTfh cell subsets, CD21^low^ B cells and switched memory B cells were collected from venous blood samples obtained concurrently with those for CXCL13 from each participant, as described below. Samples were collected in EDTA tubes and stained within 48 h (storage at RT). A 200 µl aliquot of whole blood from each participant was stained with a mixture of the following antibodies at an optimal concentration (previously determined): CCR6-FITC (Biolegend), ICOS–PE (Beckman Coulter), CD62L-PerCP-eFluor710 (eBiosciences), PD-1-PE/CY7 (Beckman Coulter), CXCR5-APC (R&D Systems), CD3-APC/A700 (Beckman Coulter), CD45RA -APC/A750 (Beckman Coulter), CXCR3-BV421 (BD Biosciences), CD4-Krome Orange (Beckman Coulter). Samples were incubated at 2–8 °C for 30 min in the dark. IOTest 3 10X Fixative Solution (Beckman Coulter) and Versalyse Lysing solution (Beckman Coulter), previously mixed as per manufacturer instructions, were added to the samples, followed by a 10 min incubation at RT protected from light. The samples were then centrifuged at 2000 RPM for 5 min, and the supernatant was removed by aspiration. A minimum of 5 × 10^4^ CD3^+^ T cells were acquired with the Gallios flow cytometer (Beckman Coulter) and analysed with Kaluza software version 1.5 (Beckman Coulter). cTfh cells were defined as CXCR5^+^CD4^+^CD45RA^−^ cells [39, 49]. The combination of the chemokine receptors CXCR3 and CCR6 was used to distinguish the three major subsets within cTfh cells: cTfh1 cells (CXCR3^+^CCR6^−^), cTfh2 cells (CXCR3^−^CCR6^−^) and cTfh17 cells (CXCR3^−^CCR6^+^) [39, 49]. Furthermore, the frequencies for the subgroups of each of the three major cTfh cell subsets (ICOS^+^PD-1^+^ and ICOS^−^PD-1^+^) were determined (Figure S1, supplementary material) [50].

### Sample Collection, Staining and Flow Cytometry for Measurement of CD21^low^ and Switched Memory B Cell Frequencies

Similarly, measurement of the frequencies of CD21^low^ B cells and switched memory B cells as per the EUROclass classification was performed at the diagnostic immunology laboratories of the Departments of Immunology at University Hospital of Wales in Cardiff and King’s College Hospital in London. Briefly, peripheral blood mononuclear cells from whole blood, obtained and processed as described above, were stained for 20 min at 4 °C with a mixture of the following antibodies at optimal concentrations (previously determined): CD45-Krome Orange (Beckman-Coulter), CD19-ECD (Beckman-Coulter), IgD-FITC (Biolegend), IgM-PB (Beckman-Coulter), CD27-PC7 (Beckman-Coulter), CD38-APC/A750 (Beckman-Coulter), and CD21-PE (BD Biosciences) [8]. Data acquisition was performed with the Gallios flow cytometer (Beckman Coulter) and analysed with Kaluza software version 1.5 (Beckman Coulter). Switched memory B cells were defined as CD19^+^IgD^−^IgM^−^CD27^+^CD38^+/−^ cells [51]. Low switched memory B cells category (smB-) was defined according to criteria described by the EUROclass classification study and considered as having ≤ 2% switched memory B cells [8]. Likewise, CD21^low^ B cells were considered high when constituting ≥ 10% B cells [8].

### Statistics

Multiple regression analysis was performed using STATA software. The rest of the statistical analysis was performed via GraphPad Prism software. The results for the CXCL13 levels were compared between the CVID and healthy control populations using the Mann-Whitney test for non-parametric data. Comparison between 2 values of CXCL13 from the same individual was carried out with Wilcoxon paired analysis. The analysis of repeated measurements of CXCL13 was evaluated by Friedman non-parametric test. For correlation analysis, the non-parametric Spearman test was used. False discovery rate (FDR) was controlled using the Benjamini-Hochberg procedure to adjust p-values in multiple correlation analyses. For 2 × 2 contingency table analysis, Fisher’s exact test was used for non-parametric data. For 3 × 2, 4 × 2 etc. contingency table analysis, the chi-square test for trend was used. ROUT test was used to exclude statistical outliers. Statistical significance was considered for *p* < 0.05 (* indicates statistical significance for *p* < 0.05, ** indicates statistical significance for *p* < 0.01, *** indicates statistical significance for *p* < 0.001, **** indicates statistical significance for *p* < 0.0001, and n.s. indicates no statistical significance for *p* > 0.05).

## Results

### Serum Levels of CXCL13 in Patients with CVID Compared to Healthy Donors

**Fig. 1:**
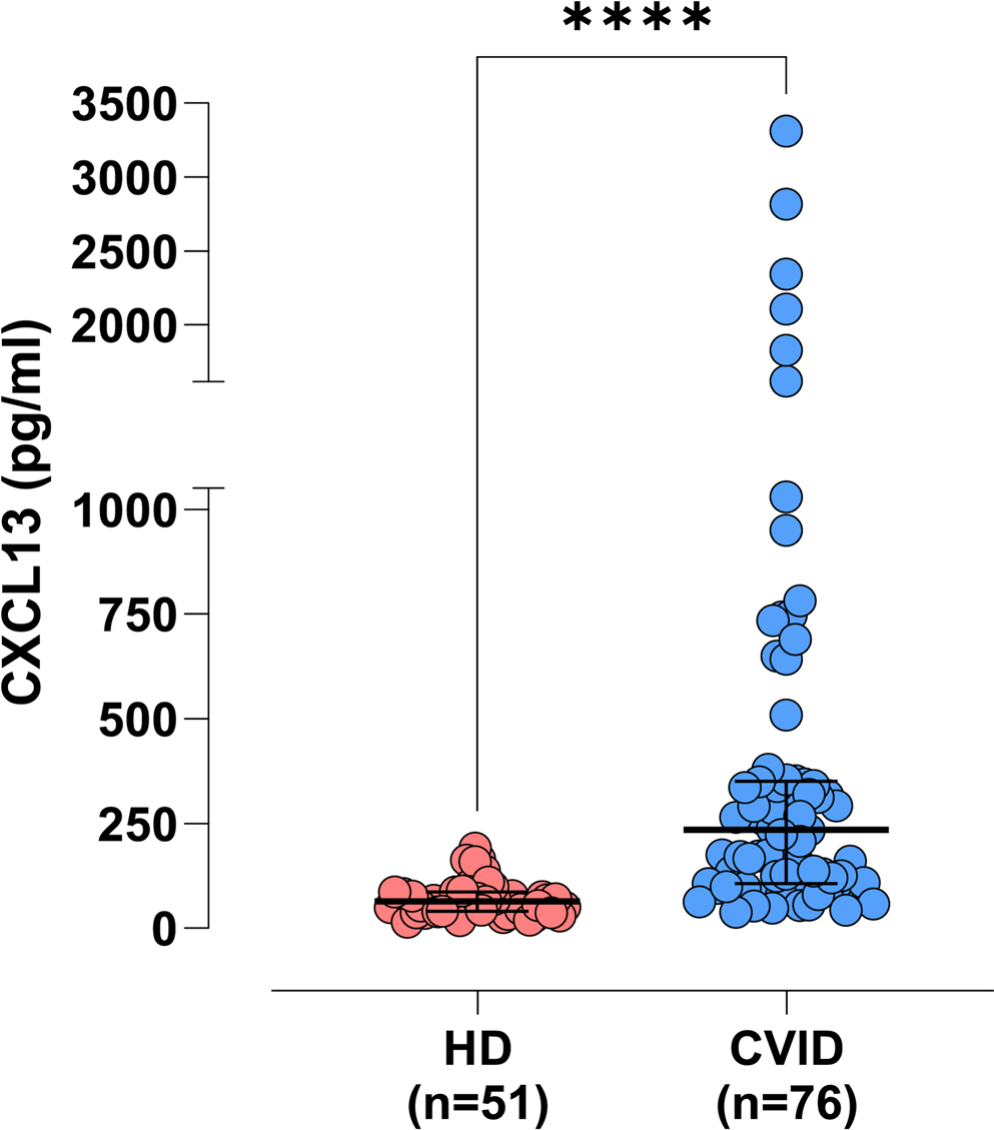
“Comparison between serum CXCL13 levels in CVID vs HD. Horizontal lines represent the median values of CXCL13, and error bars represent 75th and 25th percentiles, respectively. Y axis was divided into 2 segments for visualisation purposes. CVID: Common Variable Immunodeficiency; HD: healthy donors; n: number of individuals tested. Data were statistically analysed by the Mann-Whitney non-parametric test (**** statistical significance for *p* < 0.0001, * statistical significance for *p* < 0.05)

**Fig. 2:**
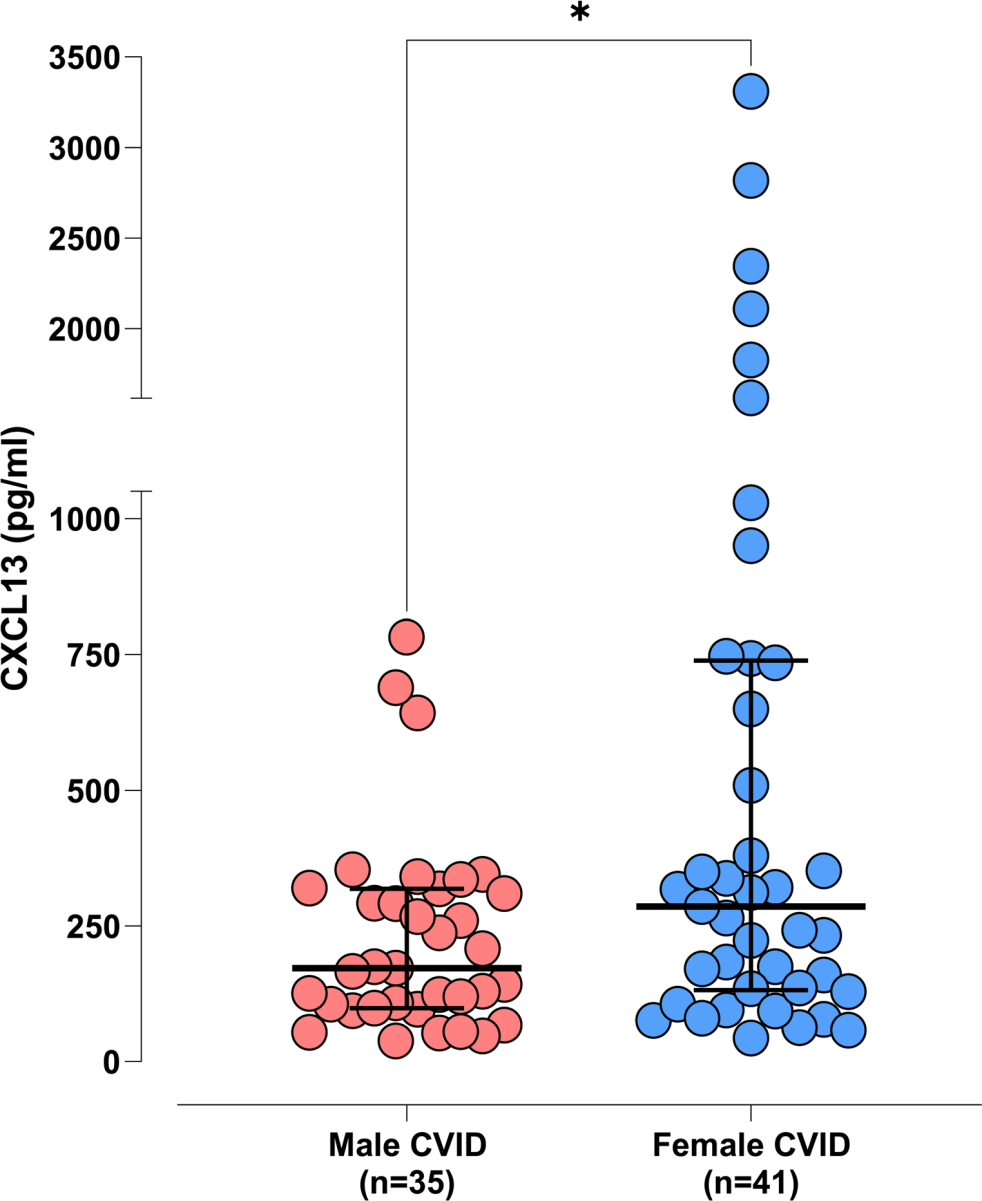
Comparison between serum CXCL13 levels in male vs. female patients with CVID. Horizontal lines represent the median values of CXCL13, and error bars represent 75th and 25th percentiles, respectively. Y axis was interrupted into 2 segments for visualisation purposes. CVID: Common Variable Immunodeficiency; n: number of individuals tested. Data were statistically analysed by the Mann-Whitney non-parametric test (* statistical significance for *p* < 0.05)

We measured serum CXCL13 in 51 healthy individuals (HD) and 76 patients with CVID. The median serum CXCL13 for CVID patients (235.40 pg/ml) was significantly higher than the median for healthy controls (76 pg/ml, Fig. 1). This significance persisted even after removing the statistical outliers (Figure S2, supplementary material). The median serum CXCL13 value for female patients (285.60 pg/ml) was significantly higher (*p* < 0.05) than the median for male patients (172.30 pg/ml, Fig. 2). The above contrasts with the levels of CXCL13 in HD where there was no statistically significant difference between males and females (see supplementary material, Figure S3). By removing 9 statistical outliers (2 males and 7 females with the highest CXCL13 values) there was no statistically significant difference (*p* = 0.17) between the medians of the 2 genders in our CVID cohort (see supplementary material, Figure S4). Additionally, patients with CVID had higher levels of CXCL13 than their sex- and age-matched healthy peers (Figure S5, supplementary material). Age did not seem to influence the levels of CXCL13 (Figure S6a, supplementary material).

### Consistency in the Levels of CXCL13 Across Repeated Measurements in Patients with CVID

**Fig. 3:**
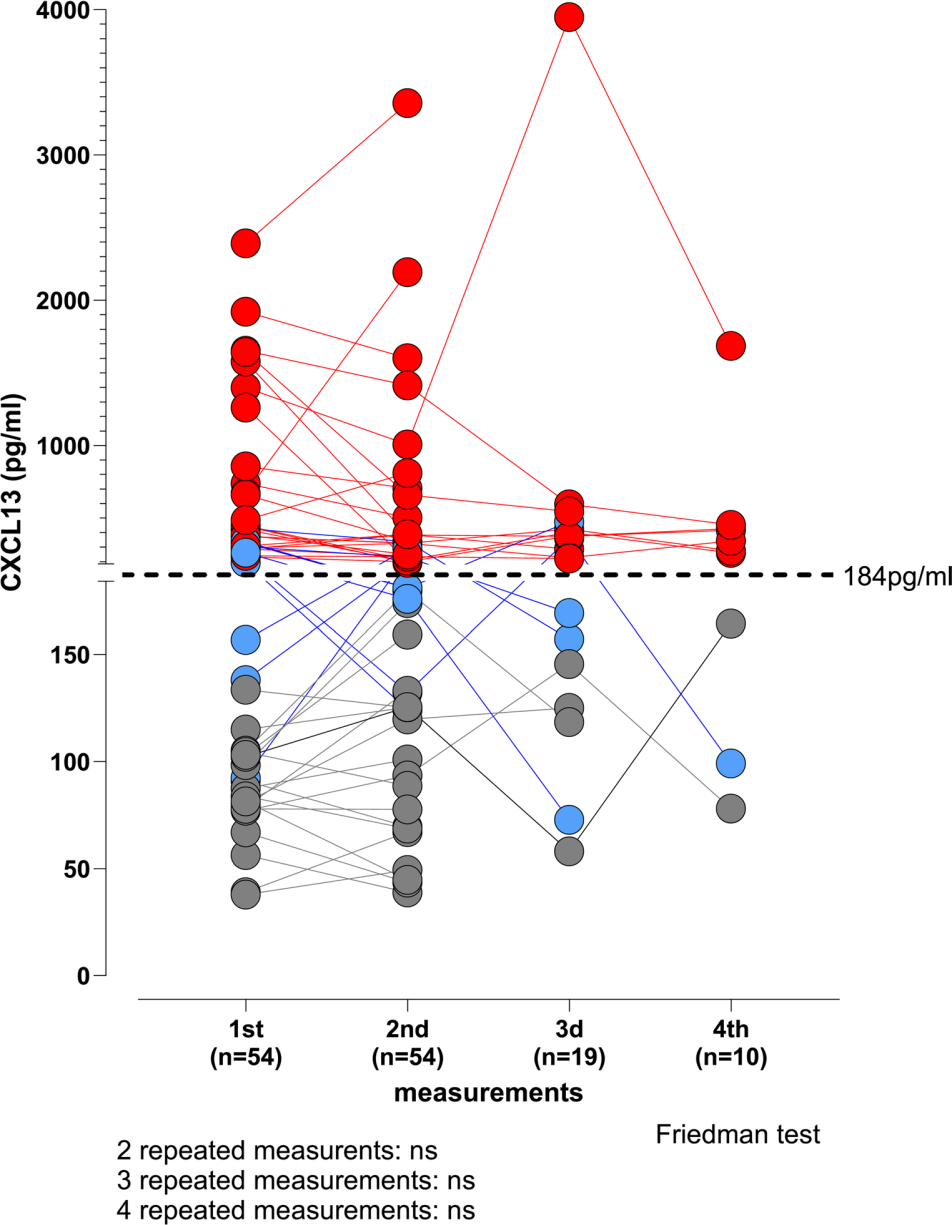
The change of CXCL13 values over time in sera of 54 patients with CVID having repeated measurements. 3 consecutive measurements were available in 19, and 4 consecutive measurements in 10 of these patients. Measurement 1, measurement 2, measurement 3 and measurement 4 represent the first, second, third and fourth measurements of CXCL13, respectively. Each horizontal line of the same colour joins the repeated measurements of CXCL13 values in the same individual. For visualisation purposes data-points are colour-coded by subgroup based on the levels of CXCL13 across all measurements for each individual: red (constantly high CXCL13), blue (crossing the upper reference limit of 184 pg/ml – dashed line), and grey (constantly normal CXCL13), n: number of subjects. The analysis of repeated measurements was evaluated by Friedman non-parametric test (ns indicates no statistical significance for *p* < 0.05)

Given the elevated serum CXCL13 levels in patients with CVID, we assessed their consistency across repeated measurements. Among 54 patients with a second measurement, 19 had a third and 10 had a fourth measurement at random intervals (median interval: 5 months, range: 0.5–32 months). No significant changes were observed from one measurement to subsequent ones (1st to 2nd: *p* = 0.06; 2nd to 3rd: *p* = 0.53; 3 d to 4th: *p* = 0.70), indicating temporal stability (Fig. 3). Interestingly, in 44 (82%) of the 54 patients with CVID who had repeated measurements of CXCL13, the levels remained consistently either below or above the threshold of the normal reference range of 184 pg/ml (Fig. 3).

### Association of Change in Serum CXCL13 Values with the Change of Serum CRP from Normal to Elevated Levels

Since CRP is a non-specific inflammatory marker, which may fluctuate between infections and has been found to be elevated in patients with CVID, we wondered whether changes in CRP levels influence the levels of CXCL13 in the serum of patients with CVID. We identified 13 patients who experienced changes in CRP from normal to elevated levels or vice versa and checked their respective serum CXCL13 levels. We observed that the change in CRP does not seem to influence CXCL13 levels (*p* = 0.19) in 13 patients with CVID (Figure S6b, supplementary material). Interestingly, from the 13 patients with CVID only in 1 the value of CXCL13 went from below to above the upper reference limit of CXCL13 for 184 pg/ml as derived by the HD CXCL13 range in serum between measurements.

### Weak Negative Correlation Between the Serum Levels of CXCL13 and the “Pre-replacement” Serum IgG Values Before Initiation of IgG Replacement for 57 Patients with CVID

**Fig. 4:**
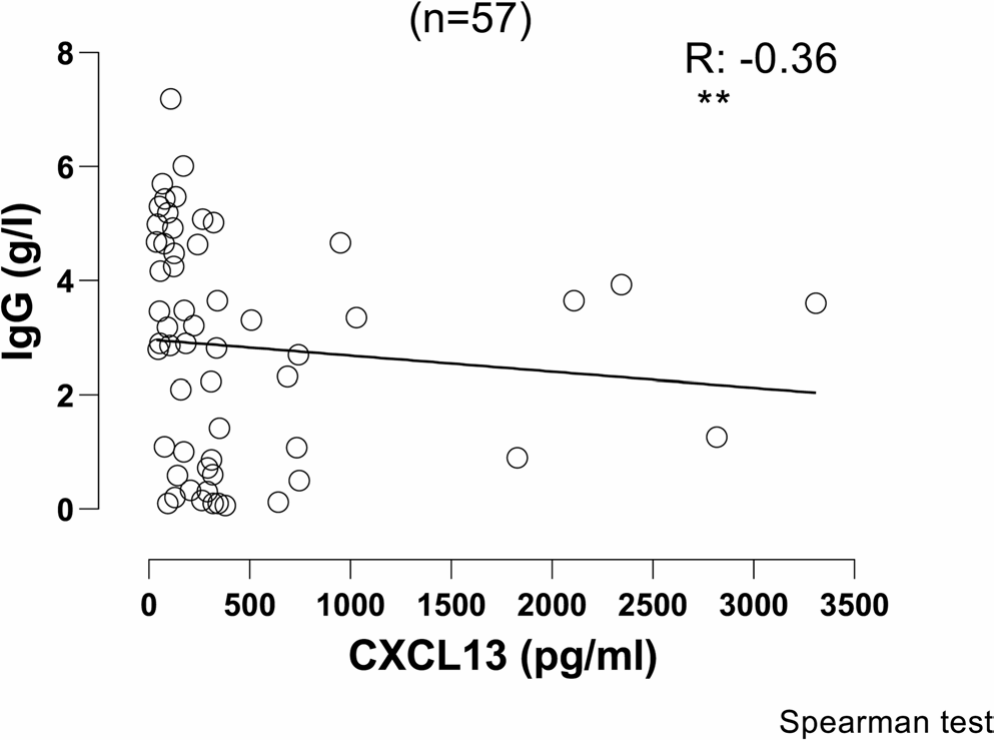
Correlation between the levels of CXCL13 in sera of 57 patients with CVID and their “pre-replacement” serum levels of IgG before the initiation of immunoglobulin replacement. R: correlation coefficient; - indicates negative correlation for R between − 1 and 0. As data did not follow a Gaussian distribution, correlation was evaluated using Spearman’s analysis. (** indicate statistical significance of the correlation for *p* < 0.01)

Since low IgG serum levels are associated with a tendency for recurrent infections and are one of the diagnostic criteria for CVID, we sought to investigate whether lower IgG levels are associated with higher levels of CXCL13 in serum. We identified 57 patients with CVID for whom we had data about their “pre-replacement” IgG levels prior to the initiation of immunoglobulin replacement and juxtaposed the levels of CXCL13 we had measured for each one of them. There was a weak negative correlation between the two parameters (*R *= −0.36, *p* = 0.006, Fig. 4).

### Immunoglobulin Replacement did not Affect the Levels of CXCL13 in 10 Patients with CVID

Since the gold standard management for symptomatic patients with CVID is immunoglobulin replacement, we wanted to investigate whether this intervention may alter the levels of CXCL13. We measured the levels of CXCL13 in 10 patients with CVID at 2 different time points, before and after the initiation of immunoglobulin replacement (median time: 5 months; range: 1–16 months). We observed that the change in CXCL13 was not statistically significant (*p* = 0.43, Figure S6c, supplementary material). Notably, CXCL13 levels in the two patients with very high baseline values remained within the “high” range, despite their drop after IgG replacement therapy. However, the small sample size limits our ability to draw any definitive conclusions.

### Correlation Between Serum CXCL13 Values and Concurrent β2-Microglobulin Levels in Patients with CVID

**Fig. 5:**
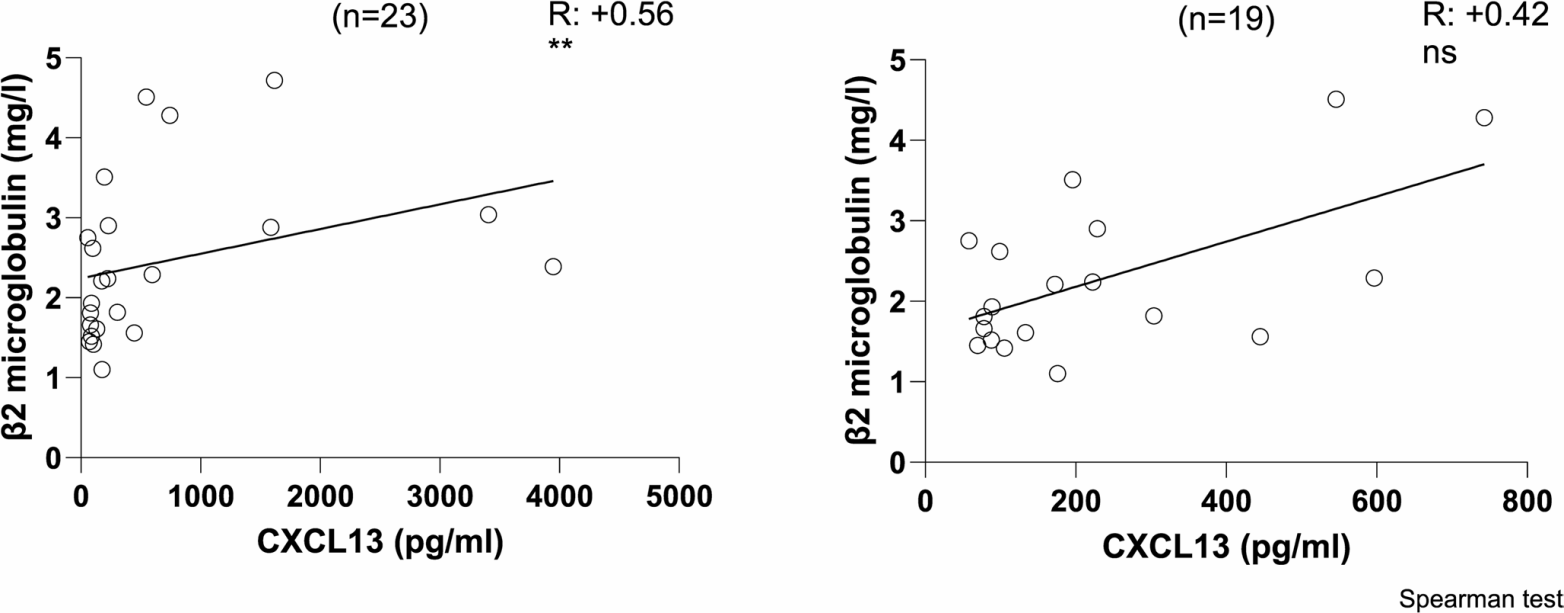
Correlation between CXCL13 values in sera of CVID patients and their corresponding values of β2 microglobulin. (Left) Correlation between CXCL13 values in sera of 23 CVID patients and their corresponding values of β2 microglobulin. (Right) Correlation between CXCL13 values in sera of 19 CVID patients and their corresponding values of β2 microglobulin after having removed the 4 statistical outliers for CXCL13. R: correlation coefficient; + indicates positive correlation for R between 0 and 1. n: number of CVID patients. The correlation was evaluated using Spearman’s non-parametric analysis. (** indicate statistical significance of the correlation for *p* < 0.01, ns: no statistical significance for *p* < 0.05)

Given the raised serum CXCL13 and β2-microglobulin levels in patients with CVID, we investigated whether serum CXCL13 levels correlated with β2-microglobulin levels. We observed that serum CXCL13 levels in 23 patients with CVID were positively correlated (R = + 0.56, *p* = 0.005) with β2-microglobulin levels (Fig. 5). Four of our patients had extremely high values of CXCL13. Therefore, we explored whether these 4 values drove the statistical significance of the positive correlation between CXCL13 and β2-microglobulin. We statistically confirmed the 4 outliers in the CXCL13 values (ROUT analysis). Following outlier removal, serum CXCL13 levels in 19 patients with CVID are positively correlated (R = + 0.42) with β2-microglobulin levels, but this correlation was not statistically significant (Fig. 5).

### Correlation Between the Number of Complications and Serum CXCL13 Levels in Patients with CVID

**Fig. 6:**
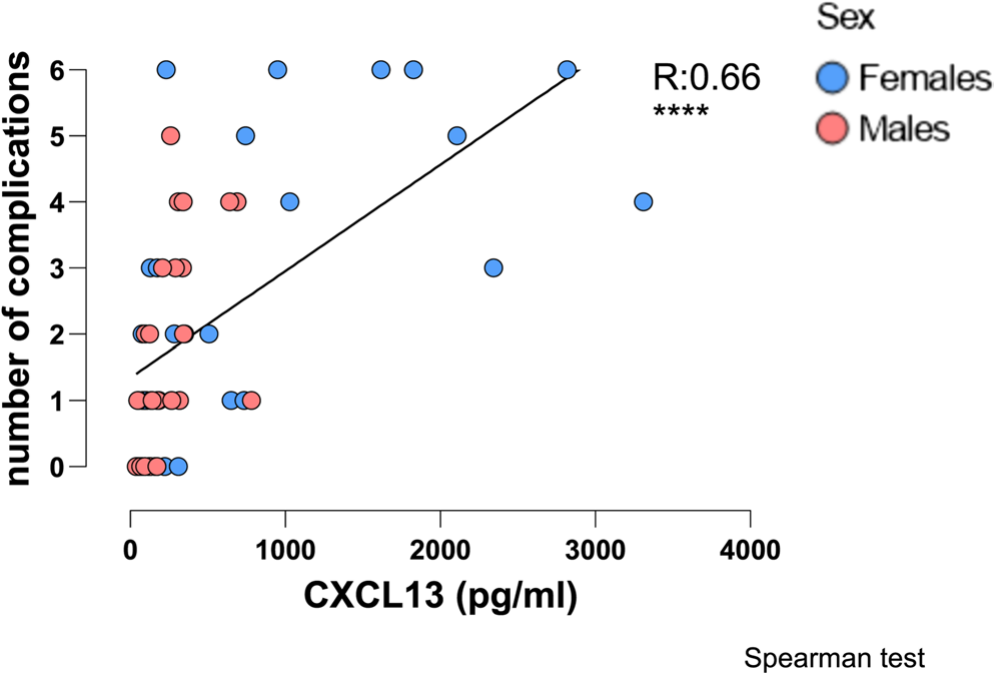
Correlation between CXCL13 values in sera of patients with CVID and their corresponding number of complications. Correlation between CXCL13 values in sera of 59 CVID patients and their corresponding number of complications. R: correlation coefficient; + indicates a positive correlation for R between 0 and 1. The correlation was evaluated using Spearman analysis. (*** indicate statistical significance of the correlation for *p* < 0.001, **** statistical significance for *p* < 0.0001)

In light of the elevated serum CXCL13 in patients with CVID, we wondered if CXCL13 levels are related to the clinical phenotype. Therefore, we enumerated the number of complications (different individual diagnoses) in each patient and examined the relationship between these numbers and serum CXCL13. Serum CXCL13 is strongly correlated with the number of complications in CVID (R = + 0.70, *p* < 0.0001, Fig. 6). Although the average number of complications was indeed higher in females than in male patients, we did not identify any statistical difference between the two genders for the number of complications (Figure S7, supplementary material). Both sexes showed a positive correlation between CXCL13 and the number of complications (Figure S8, supplementary material).

### Comparison of CXCL13 Levels Between Patients with CVID With or Without Complications

**Fig. 7:**
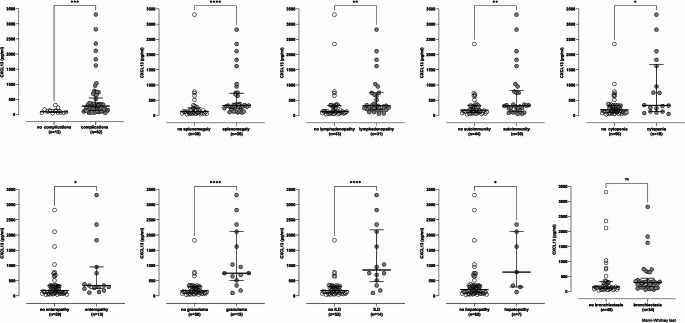
Comparison between the serum levels of CXCL13 in patients with CVID with and without complications. Middle horizontal lines represent median values of CXCL13, upper horizontal lines represent 75th percentile and lower horizontal lines 25th percentile. Data were statistically analysed by the Mann-Whitney non-parametric test. (* indicates statistical significance for *p* < 0.05, ** statistical significance for *p* < 0.01, *** indicate statistical significance for *p* < 0.001, **** statistical significance for *p* < 0.0001, and ns indicates no statistical significance for *p* < 0.05)

We then compared the levels of CXCL13 in patients with CVID with or without given complications. Overall, patients with CVID, with at least one complication, had higher levels of CXCL13 than those with no complications (*p* = 0.0002; Fig. 7). Except for bronchiectasis, patients with CVID with known complications had higher levels of CXCL13 than those without complications. The complications investigated included splenomegaly (p = < 0.0001), lymphadenopathy (*p* = 0.001), autoimmunity (*p* = 0.007), cytopenia (*p* = 0.02), enteropathy (*p* = 0.01), hepatopathy (0.01), interstitial lung disease (ILD) (*p* < 0.0001) and granulomata (*p* < 0.0001).

### Association of Serum CXCL13 with Granulomatous Formation in CVID

Since higher CXCL13 levels have been associated with the presence and number of non-infectious complications in CVID, we performed a multiple linear regression analysis to determine which CVID complication is most strongly associated with levels of CXCL13, adjusting for gender and all other complications. For patients with repeated CXCL13 measurements, the mean value per patient was used. Given the limited sample size (*n* = 76), we restricted the number of parameters to ensure model stability and an appropriate ratio of predictors to observations. The model included six complications as independent variables: splenomegaly, lymphadenopathy, granulomatosis, autoimmunity, cytopenia, and enteropathy. These were selected based on their biological relevance and sufficient representation within the cohort to allow meaningful analysis. Hepatopathy and GLILD were excluded due to their substantial clinical overlap with granulomatous disease in our cohort, to reduce collinearity and overfitting (Table 1). Prior to model inclusion, we assessed pairwise correlations between complications and confirmed that no statistically significant strong collinearity was present (after Benjamini-Hochberg FDR multiple comparison adjustments), supporting their inclusion in a single model (Supplementary Table 3). The multiple regression analysis revealed a significant positive association between CXCL13 levels and the presence of granulomata (*p* < 0.0001, Table 2), while gender did not appear to play a role.

**Table 2:** Multiple regression analysis of serum CXCL13 versus clinical complications in CVID adjusted for gender

CXCL13	Coefficient	Standard error	*p*-value (significance)	[95% confidence interval]	
Splenomegaly	173.14	171.33	0.317	−170.66	516.93
Lymphadenopathy	−173.78	191.02	0.367	−557.09	209.53
Granulomata	804.58	187.40	**< 0.0001**	428.54	1180.62
Autoimmunity	155.83	160.68	0.337	−166.60	478.27
Cytopenia	295.52	197.88	0.141	−101.56	692.59
Enteropathy	241.57	169.80	0.161	−99.16	582.29
Gender	220.69	152.60	0.154	−85.52	526.91

### Association Between Serum Levels of CXCL13 and Immune Phenotype of Patients with CVID

High frequency of CD21^low^ B cells (≥ 10% B cells) and low frequency of switched memory B cells (≤ 2% switched memory B cells) have been associated with a complex phenotype in CVID according to the EUROclass classification. Therefore, we aimed to investigate whether there is an association between the levels of CXCL13 and the corresponding frequencies of CD21^low^ B cells and switched memory B cells in patients with CVID. We identified a non-significant weak positive correlation of CXCL13 to CD21^low^ B cells (R = + 0.25, *p* = 0.13) and a non-significant weak negative correlation to switched memory B cells (*R*=−0.29, *p* = 0.08; Figure S10, supplementary material).

Subsequently, we sought to investigate whether there is a difference in the serum levels of CXCL13 in patients with CVID: (a) between those with normal frequencies of CD21^low^ B cells (CD21^norm^) and those with high frequencies of CD21^low^ B cells (CD21^low^), and (b) between those with normal frequencies of switched memory B cells (smB+) and those with low frequencies (smB-). We categorised our cohort of patients with CVID based on their frequencies of CD21^low^ B cells and switched memory B cells. We compared the CD21^norm^ group of patients with CVID to the CD21^low^ group and the smB + group with the smB- group.

**Fig. 8:**
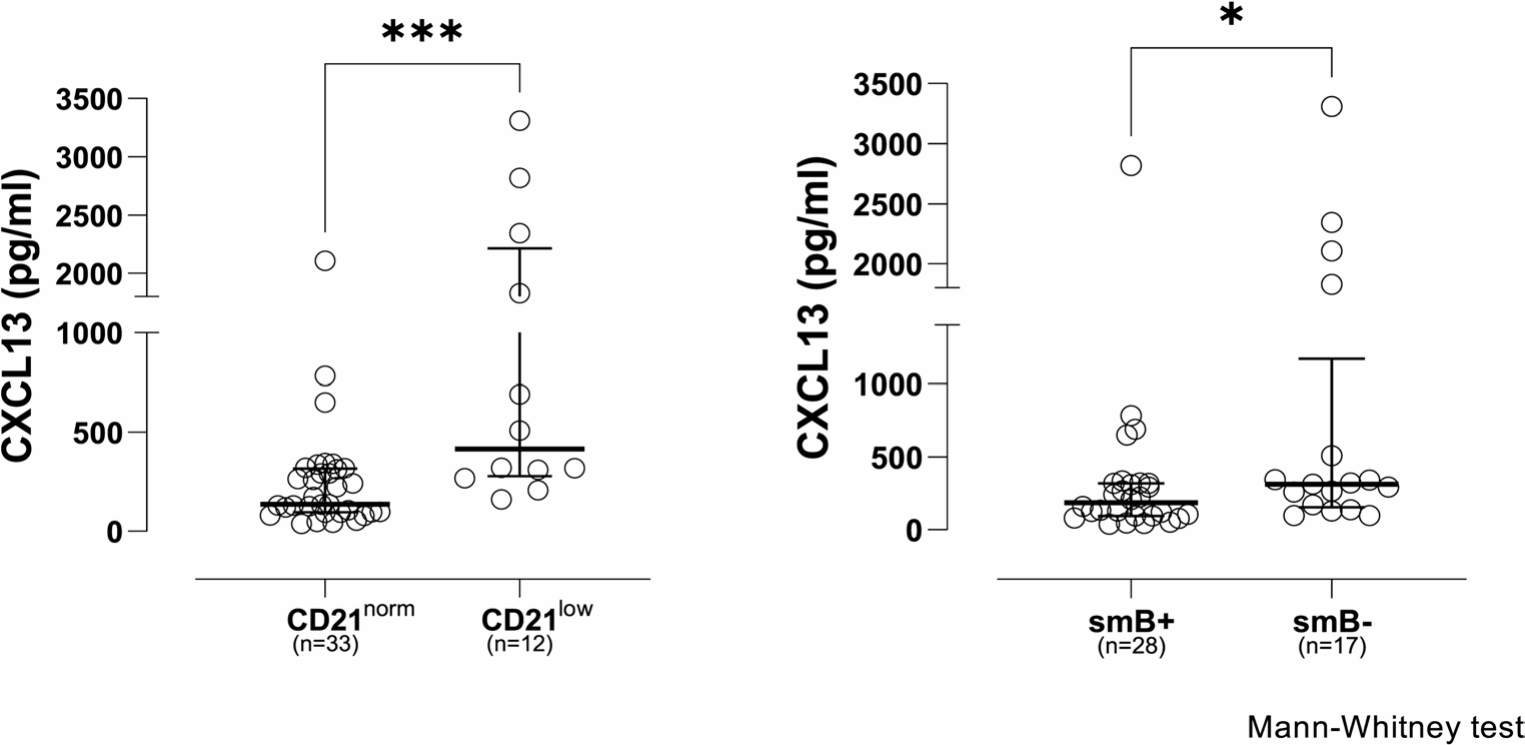
CXCL13 levels in serum compared between groups of CVID patients as stratified based on their frequencies of CD21^low^ B cells and switched memory B cells. Samples of serum from CVID patients were analysed for CXCL13 levels (see materials and methods) and the results were compared between the following groups: (a) CD21^norm^ and CD21^low,^ and (b) smB + and smB-. Horizontal lines represent the median values of CXCL13, and error bars represent 75th and 25th percentiles, respectively. Y axis was interrupted into 2 segments for visualisation purposes. CD21^norm^: CD21^low^ B cells < 10% of B cells; CD21^low^: CD21^low^ B cells ≥ 10% of B cells; smB+: switched memory B cells > 2% of B cells; smB-: switched memory B cells ≤ 2% of B cells; n: number of serum samples from different individuals. Data were statistically analysed by the Mann-Whitney non-parametric test (*** statistical significance for *p* < 0.001, * statistical significance for *p* < 0.05)

The median serum CXCL13 for the CD21^low^ group (415 pg/ml) was significantly higher (*p* < 0.001) than the median for the CD21^norm^ group (136.10 pg/ml). The minimum CXCL13 value in the former group was 159.80 pg/ml and the maximum was 3310 pg/ml. Among the latter group, the minimum value was 38.14 pg/ml and the maximum 2109 pg/ml (Fig. 8).

The median serum CXCL13 for the smB- group (311.80 pg/ml) was significantly higher (*p* < 0.05) than the median for the smB + group (183.90 pg/ml). The minimum CXCL13 value in the former group was 96.55 pg/ml and the maximum was 3310 pg/ml. The latter group’s minimum value was 38.14 pg/ml and the maximum 2818 pg/ml (Fig. 8).

### Frequency of Peripheral cTfh Cell Subsets in CVID Patients and Healthy Controls

We compared the patients with CVID and the healthy controls for their peripheral cTfh cell subsets frequency. Overall, patients with CVID had a higher frequency of total circulating Tfh cells (cTfh) compared with healthy controls (*p* = 0.0002, Table S4, Figure S11, supplementary material). In terms of cTfh cell subsets, patients with CVID have a higher frequency of cTfh1 (*p* < 0.0001) and a lower frequency of cTfh2 (*p* = 0.0018) and cTfh17 (*p* = 0.0014) compared with healthy controls.

Interestingly, patients with CVID have a higher frequency for all cTfh cell subsets expressing both PD1 and ICOS or only PD1, compared with healthy controls: PD1 + ICOS + cTfh1 (*p* < 0.0001), PD1 + ICOS + cTfh2 (*p* < 0.0001), PD1 + ICOS + cTfh17 (*p* = 0.0034), PD1 + ICOS-cTfh1 (*p* = 0.003), PD1 + ICOS- cTfh2 (*p* < 0.0001) and PD1 + ICOS- cTfh17 (*p* = 0.0159).

### Correlation Between Serum CXCL13 Values and cTfh Cell Subsets Frequency in Patients with CVID

Given the raised serum CXCL13 values, increased frequency of total peripheral cTfh cells in patients with CVID and the fact that cTfh cells are major producers of CXCL13, we wondered whether serum CXCL13 levels correlated with the frequency of peripheral cTfh cell subsets.

We observed that serum CXCL13 levels in patients with CVID were positively correlated (R = + 0.50, *p* = 0.005) with total peripheral cTfh cells (Table 3). In terms of cTfh cell subsets, we observed that serum CXCL13 levels had a positive correlation with cTfh1 cells (R = + 0.50, *p* = 0.003) and also with the PD1 + ICOS + cTfh1 (r = + 0.55, *p* = 0.001), PD1 + ICOS + cTfh2 (R = + 0.30, *p* = 0.08) and PD1 + ICOS + cTfh17 cells (R = + 0.55, *p* = 0.001). In contrast, serum CXCL13 levels had a negative correlation with cTfh2 (*R*=−0.33, *p* = 0.06) and cTfh17 (*R*=−0.47, *p* = 0.006) cells. Interestingly, there was no statistically significant correlation between serum CXCL13 values and cTfh cell subsets frequency in healthy controls, i.e. total peripheral cTfh cells (*R*=−0.17, *p* = 0.34), cTfh1 cells (*R*=−0.34, *p* = 0.52), PD1 + ICOS + cTfh1 cells (*R*=−0.20, *p* = 0.24), cTfh2 cells (*R*=−0.07, *p* = 0.67), PD1 + ICOS + cTfh2 (R = + 0.09, *p* = 0.6), cTfh17 cells (R = + 0.30, *p* = 0.07) and PD1 + ICOS + cTfh17 (*R*=−0.03, *p* = 0.87).

**Table 3:** Comparison of correlation between serum CXCL13 and cTfh cell subsets in CVID and HD

	CVID			HD		
CXCL13 correlation with cTfh subsets	*R*	*p*-value	Adj. *p*-value (FDR)	*R*	*p*-value	Adj. *p*-value (FDR)
cTfh	+ 0.50†	0.005	**0.01**	−0.17††	0.34	0.43
cTfh1	+ 0.50	0.003	**0.01**	−0.34	0.05	0.18
cTfh2	−0.33	0.06	0.10	−0.07	0.67	0.67
cTfh17	−0.47	0.006	**0.01**	+ 0.30	0.07	0.18
PD1^+^ICOS^+^cTfh1	+ 0.55	0.001	**0.01**	−0.20	0.24	0.40
PD1^+^ICOS^+^cTfh2	+ 0.30	0.08	0.11	+ 0.09	0.60	0.67
PD1^+^ICOS^+^cTfh17	+ 0.55	0.001	**0.01**	−0.03	0.87	0.87
PD1^+^ICOS^−^cTfh1	+ 0.43	0.01	**0.02**	−0.10	0.58	0.67
PD1^+^ICOS^−^cTfh2	+ 0.58	0.0005	**0.01**	−0.10	0.57	0.67
PD1^+^ICOS^−^cTfh17	+ 0.38	0.03	**0.03**	−0.13	0.40	0.53

R: Spearman correlation coefficient; †, +: positive correlation for R between 0 and 1; ††, -: negative correlation for R between − 1 and 0. Spearman analysis: statistical significance for *p* < 0.05. To account for multiple comparisons (*n* = 10 per group), p-values were adjusted using the Benjamini-Hochberg false discovery rate (FDR) method. Statistically significant adjusted p-values ≤ 0.05 are shown in bold

## Discussion

CVID is a heterogeneous disease for which few good biomarkers of poor prognosis have previously been identified [8, 11, 13, 15, 16, 52, 53]. We sought to investigate whether there is altered production of CXCL13 in patients with CVID and whether its levels in serum may help categorise this heterogeneous group of patients.

We identified that the levels of CXCL13 were higher in the sera of patients with CVID compared to the healthy donors (Fig. 1). This is in accordance with recent similar findings by Hultberg et al. [54].

One might argue that the elevation of CXCL13, a potent B-cell chemoattractant, in CVID, a condition with poor antibody formation and response, seems contradictory.

One explanation could be that the scarcity of switched memory B cells and IgG/IgA production in CVID, triggers elevated CXCL13 as an ineffective compensatory attempt to recruit B cells to antigenic sites and restore antibody production [17, 55]. This explanation is unlikely because the levels of CXCL13 in X-linked agammaglobulinemia (XLA) patients, a condition characterised by antibody failure, are similar to those of healthy donors (Figure S12, supplementary material) [44, 56].

Another explanation could be that CXCL13 reflects the immune dysregulation and chronic inflammation in complex CVID, as evidenced by the abundance of hyperplastic yet inefficient germinal centres with the expansion of cTFh cells, first described by Unger et al. and then by Romberg et al. [57, 58]. The positive correlation we identified between serum CXCL13 and cTfh cells may reflect this phenomenon. Additionally, the association of CXCL13 levels with complications of immune dysregulation in CVID, as well as the closer association with granulomatous formation in our cohort, may corroborates the above explanation. Furthermore, the higher levels of serum CXCL13 in CVID patients with splenomegaly, ILD and granulomata may suggest a link between CXCL13 and ectopic germinal centre formation and lymphopoiesis [59, 60].

The positive association between serum CXCL13 levels and cTfh cells—particularly cTfh1 subsets and those expressing the PD-1 and/or ICOS activation markers— could imply that elevated CXCL13 may result from the expansion of these populations [35, 36, 38, 39, 41, 61, 62]. This aligns with findings by Milardi et al., who reported similar associations in CVID patients with immune dysregulation-related complications such as splenomegaly and lymphadenopathy [63].

Since CXL13 is elevated in infective processes we wondered whether elevation in CVID might reflect this [28, 64–67]. However, all blood samples were collected from patients with no symptoms or signs of infection. High levels of CRP - an inflammatory marker - have been described in CVID [11, 68]. Nevertheless, we observed that in CVID patients where CRP levels fluctuated between normal and high – likely due to transient viral or bacterial infection - CXCL13 levels did not change significantly, remaining either in the “normal” or “elevated” category. Thus, elevated CXCL13, may rather reflect the chronic inflammatory process in CVID, irrespective of the presence of acute infection. To further characterise elevated CXCL13 levels in CVID, we assessed the potential influence of gender, age, baseline IgG levels, and β2-microglobulin. Female patients exhibited higher median CXCL13 levels than males (Fig. 2), a difference not observed in healthy controls (Figure S3, supplementary material). This may reflect a higher frequency of autoimmune complications among females, consistent with known female predominance in autoimmune conditions and supported by our cohort data (Table 1) [69]. However, this gender difference disappeared after excluding outlier female patients with the highest CXCL13 levels and complication burden (Figs. 6 and S4), and adjusting for gender did not alter the association between CXCL13 and CVID-related complications (Table 2). Thus, elevated CXCL13 may instead reflect heightened inflammatory activity, possibly due to granulomatous disease or cumulative complications, rather than gender alone. A positive correlation between CXCL13 levels and the number of complications was observed across both sexes (Figure S8, supplementary material), suggesting gender-independent inflammatory involvement. Whether, within the group of patients with complex CVID, female gender predisposes for heightened inflammatory activity and therefore higher CXCL13 levels would be interesting to explore in a larger-scale study.

Low baseline IgG levels have been associated previously with worse prognosis in patients with CVID [3]. In this study, there was a weak negative correlation between the CXCL13 levels and the lowest (pre-replacement) IgG levels in the patients with CVID before commencing immunoglobulin replacement.

β2-microglobulin a marker of immune dysregulation and turnover, as well as haematologic malignancy has previously been found to be elevated in CVID and associated with disease severity [12, 13, 70]. The positive correlation to the levels of CXCL13 in patients with CVID disappeared by omitting the 4 statistical outliers based on their extreme values of CXCL13 (Fig. 5). It would be valuable to investigate in a larger cohort whether these four with high complication burden were “driving” the observed correlation, and to assess the influence of gender, serum baseline IgG, together with β2-microglobulin through multivariable regression analysis. In our patients with CVID, the higher the serum CXCL13 value, the higher the number of complications (Fig. 6). Furthermore, patients suffering from any specific complication (except bronchiectasis) had a higher serum CXCL13 level compared with patients without that particular complication (Fig. 7). It has already been shown that patients with non-infectious complications were significantly more likely to have decreased survival than those with infections only [3]. Taken together, this suggests that measuring serum CXCL13 may enable the detection of complications, thus facilitating their early diagnosis and treatment. Additionally, given the fact that there may be an overlap of these in an individual, our results suggest that higher levels of CXCL13 may better indicate the existence of granulomata rather than other specific complications (Table 2, supplementary material). The influx of B and T cell populations, together with the beneficial therapeutic effect of B cell depletion by Rituximab in CVID-related granulomata, may be a manifestation of an essential functional role of CXCL13 in the pathology of granulomata in CVID [71, 72]. Perhaps, further studies using immunohistochemistry to detect CXCL13 in tissues affected by CVID complications could provide definitive answers regarding its cellular origin.

EUROclass trial showed that increased CD21^low^ B cells were associated with the presence of splenomegaly and reduced switched memory B cells with splenomegaly and granulomata [8]. The association of higher CXCL13 with patients having high CD21^low^ B cell or low switched memory B cell frequency and its weak correlation – although not statistically significant - with the frequency of CD21^low^ B cells and switched memory B cells corroborate the suggestion for its use as a biomarker of complex phenotype in CVID (Figs. 8 and S10, supplementary material). CXCL13 may be a more specific predictor for granulomatous disease in CVID. Furthermore, it would be intriguing to explore in a larger cohort the value of CXCL13 as a biomarker for specific complications in CVID, along with other suggested biomarkers such as IL-10 or CXCL10 [54, 73].

Since CVID is a lifelong condition, we wanted to explore whether the elevated CXCL13 levels in a patient with CVID remain constantly elevated over time or change with repeated measurements, with the change of CRP, or with medical interventions such as immunoglobulin replacement. We identified that there is categorical consistency in the levels of CXCL13 remaining within the same “normal” or “elevated” value range and not varying significantly across repeated measurements in the same individuals, regardless of CRP changes (Figs. 3 and S6b-c, supplementary material). This is consistent with CXCL13 being a reliable biomarker for patients with CVID, that is not affected by transient acute inflammatory episodes. Interestingly, 82% of the patients with CVID, having had repeated measurements of CXCL13, did not cross the CXCL13 cut-off limit of 184 pg/ml, corroborating the consistency of CXCL13 measurements and the ability to characterise a patient with CVID by a single measurement of a biomarker (Fig. 3).

Furthermore, the therapeutic mainstay for patients with CVID, namely immunoglobulin replacement, seems not to affect the levels of CXCL13, adding to the clinical usefulness and reliability of measuring CXCL13 [74]. This is supported by the fact that the 7 patients with XLA – all under immunoglobulin replacement - at a single CXCL13 measurement had serum levels similar to the healthy donors and not within the “elevated” range. Consistently high levels of CXCL13 may indicate the presence of immune dysregulation and chronic inflammation in CVID. Notably, no patients on immunosuppressive treatment were included in this study, and therefore the effect of such treatments on CXCL13 serum levels cannot be determined.

Limitations of the study were the following: Patients with CVID suffer from recurrent infections, and this prevented some from attending their clinical appointments or having clinical investigations; hence, some of the clinical information is missing, and it was not possible to standardise the time for receiving repeated samples. Also, part of the study was conducted during the COVID-19 pandemic, when access to the hospital for these patients was hugely restricted. Patients with CVID constitute a relatively rare population, and recruiting them can be challenging, so the sample is small. This was an exploratory study presenting the results from the cohort of CVID patients in a single centre who consented to participate. To confirm these and generalise would require a comprehensive survey from a subsequent multi-centre study.

Samples for repeated measurements were obtained from patients receiving intravenous (as opposed to subcutaneous) immunoglobulin replacement as it was more convenient due to their frequent hospital visits to receive their infusions. For the multiple regression analysis, we did not include hepatopathy and ILD as there was significant overlap with the granulomata. Finally, we were not given exact values for normal levels of CRP below 2 mg/dl, but these were reported as < 2 mg/dl. So, we could not perform a correlation analysis of the exact CRP values with the corresponding CXCL13 ones.

In conclusion, CXCL13 appears to be a useful marker for categorising heterogeneous patients with CVID into those without immune dysregulation-related complications (“normal” serum CXCL13) and those with a complex clinical and immune phenotype (“high” serum CXCL13).

We propose CXCL13 as a biomarker of complex phenotype in CVID, reflecting the immune dysregulation, antibody failure and increased germinal centre activity. Measuring high serum CXCL13 levels -associated with other markers of the complex phenotype (expanded CD21^low^ B cells, low switched memory B cells, high β2-microglobulin and low baseline IgG levels) - may suggest the presence of complications, thus allowing for enhanced monitoring and potentially early intervention [3, 8, 9, 13, 14]. In the era of personalised therapeutic management, our study, as has happened with the Tfh cells, may help pave the way for exploring therapeutic options targeting CXCL13 in patients with complex CVID [40]. A more extensive multi-centre study is required to confirm our findings.

## Supplementary Information

Below is the link to the electronic supplementary material.

Supplementary Material 1 (DOCX 1.77 MB)

## Data Availability

Not applicable.
