# Supplementary material for: CXCL13 as a Biomarker of Complex Common Variable Immunodeficiency
Source: J Clin Immunol. 2025 Nov 25;45(1):168. doi: 10.1007/s10875-025-01963-2 (PMC12647345; doi:10.1007/s10875-025-01963-2)
Supplement: Supplementary file 1 — (DOCX 1.77 MB) [file 10875_2025_1963_MOESM1_ESM.docx]

**SUPPLEMENTARY MATERIAL**

**SUPPLEMENTARY MATERIAL**

**Demographics of CVID patients and HD participating in the study**

| Table S1. Demographics of participants | | |  |
| --- | --- | --- | --- |
|  | HD | CVID | p-value (significance) |
| Number of participants;  male/ female (total) | 22/29 (51) | 35/41 (76) | 0.9 (n.s.) |
| Age; min-max (mean) | 22-64 (40) | 19-76 (41) | 0.85 (n.s.) |
| Male age; min-max (mean) | 22-58 (37) | 19-76 (38) | 0.77 (n.s.) |
| Female age; min-max (mean) | 23-64 (42) | 19-74 (44) | 0.58 (n.s.) |
| CVID: Common Variable Immunodeficiency; HD: healthy donors; n.s. indicates no statistical significance for p<0.05. Data were statistically analysed by the Mann-Whitney non-parametric test. | | | |

**No statistically significant difference is found in the age between participants with CVID and HD and between the participants of the same sex from both cohorts**

There was no statistically significant age difference between the patients with CVID and the healthy volunteers (p=0.8474, Table S1). The median age for both HD and patients with CVID was 37 years. The minimum age for HD was 22 years and the maximum was 64 years. Among patients with CVID the minimum age was 19 and the maximum 76 years. The median age for females with CVID was 40 years while for female HD it was 38 years. The minimum age for female patients with CVID was 19 and the maximum 74 years, while for female HD the minimum age was 23 and the maximum 64 years. The median age for males with CVID was 32 and for male HD was 33.5 years. The minimum age for male patients with CVID was 19 and the maximum 76 years, while for the male HD the minimum age was 22 and the maximum 58 years.

**The gating strategy for identifying the peripheral cTfh cell subsets**

**
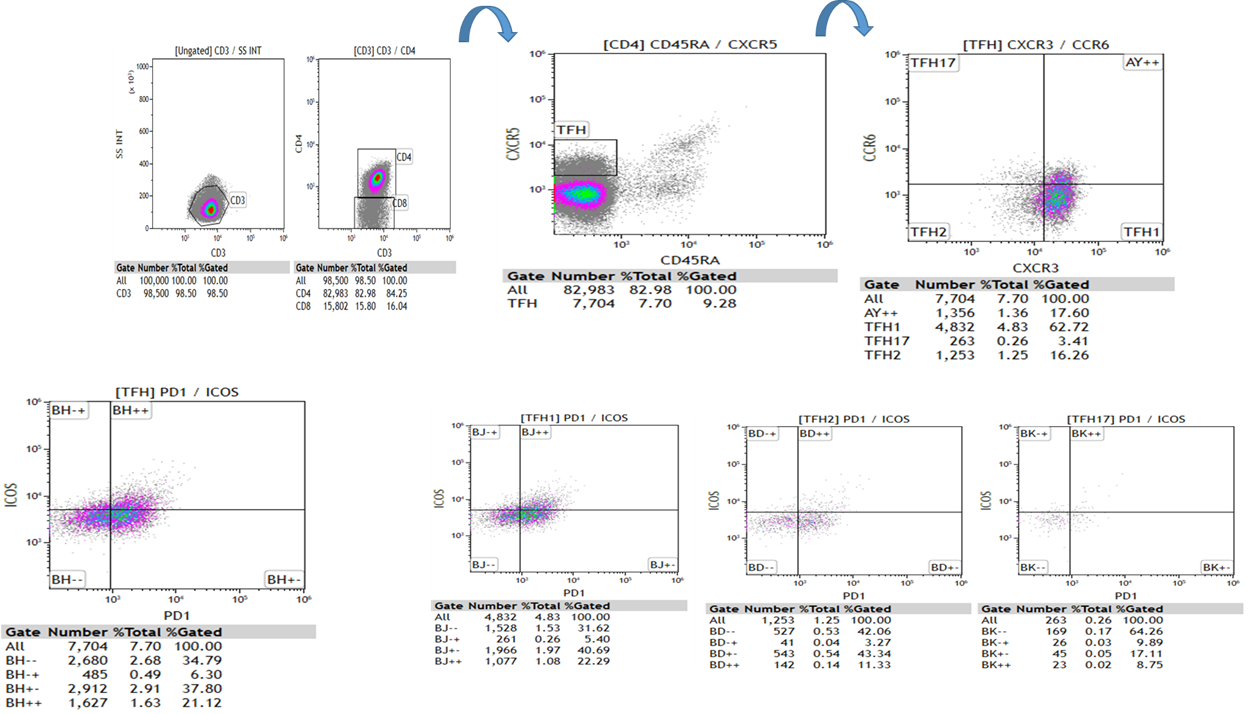
**

**Figure S1.** **Flow cytometric analysis of peripheral cTfh cell subsets.** PBMCs were analysed by flow cytometry. After gating on lymphocytes according to forward (FSC)/side scatter (SSC), T cells were characterized by CD3 staining. By staining for CD4, CXCR5 and CD45RA the total Tfh cell population was distinguished as (CD3+CD4+CXCR5+CD45RA-) cells. Further staining for CXCR3 and CCR6 distinguished the 3 major Tfh cell subsets: CXCR3+/CCR6- (Tfh1), CXCR3-/CCR6- (Tfh2) and CXCR3-/CCR6+ (Tfh17). The staining for the expression of ICOS and PD-1 allowed the additional distinction of ICOS^+^PD-1^-^ andICOS^-^PD-1^+^ subsets for each of the Tfh1, Tfh2, and Tfh17.

**Serum levels of CXCL13 in patients with CVID compared to healthy donors without the statistical outliers**

**Figure S2.** Comparison between serum CXCL13 levels in CVID vs HD without the outliers. Horizontal lines represent median values of CXCL13, error bars represent 75% and 25% percentile. Y axis was interrupted into 2 segments for visualisation purposes. CVID: Common Variable Immunodeficiency; HD: healthy donors; n: number of individuals tested. Data were statistically analysed by the Mann-Whitney non-parametric test (**** statistical significance for p<0.0001, * statistical significance for p<0.05).

**No statistically significant difference is seen in serum CXCL13 levels between the cohorts of healthy male and female individuals**

Since CXCL13 levels in serum were found to be higher in female patients with CVID compared to their male counterparts, we wondered whether there was a difference in serum CXCL13 between the male and the female HD. There was no statistically significant difference in the levels of CXCL13 in serum between male and female HD (p=0.7273, figure S3). The median serum CXCL13 value for female HD was 57.39 pg/ml and 68.85 pg/ml for male HD. The minimum CXCL13 value among female HD was 14.50 pg/ml and the maximum was 190.7 pg/ml. Among male HD the minimum value was 19.44 pg/ml and the maximum was 159.1 pg/ml.

**Figure S3.** CXCL13 levels in serum compared between male and female HD. Samples of serum from HD were analysed for CXCL13 levels (see materials and methods) and the results compared between the 2 groups. Horizontal lines represent median values of CXCL13, error bars represent 75% and 25% percentile. HD: healthy donors; n: number of serum samples from different individuals. Data were statistically analysed by the Mann-Whitney non-parametric test. Ns indicates no statistical significance of the comparison (statistical significance for p<0.05)**.**

**N****o statistically significant difference is seen in serum CXCL13 levels between the cohorts of male and female CVID patients after removing the statistical outliers**

**Figure S4.**  Comparison between serum CXCL13 levels in male vs female patients with CVID. Left: the whole CVID cohort. Middle: after having removed 9 CVID statistical outliers. Right: After having removed 8 females with the highest CXCL13 measurements. Horizontal lines represent median values of CXCL13, error bars represent 75% and 25% percentile. Y axis was interrupted into 2 segments for visualisation purposes. CVID: Common Variable

**Age-matched females and males with CVID have higher serum values of CXCL13 compared to their same sex HD counterparts**

Since serum levels of CXCL13 were found to be higher in patients with CVID compared to HD and higher among the female patients with CVID, we wondered whether there was a difference in CXCL13 between participants from both cohorts having the same sex and age. We identified 29 female patients with CVID, age-matched with 29 female HD, and 19 male CVID patients age-matched with 19 male HD. We observed that serum CXCL13 levels were significantly higher in patients with CVID when compared to same age and sex HD (age-matched female pairs: p<0.0001, age-matched male pairs: p=0.0005, figure S5).

**Figure S5.** CXCL13 levels in serum compared between a) 29 age-matched female CVID patients to 29 HD, and b) 19 age-matched male patients with CVID to 19 HD. The age-matched cohorts were compared for their levels of serum CXCL13. Each horizontal line joins the two age-matched participants i.e., one patient with CVID and one HD having the same age. The paired analysis was performed by Wilcoxon non-parametric test (statistical significance for p<0.05).

**No association was identified between the age of patients with CVID and their respective serum CXCL13 levels**

We wanted to investigate whether the serum levels of CXCL13 were associated with the age of the patients with CVID. Hence, we examined the relationship between the serum levels of CXCL13 and the respective age during venepuncture for measuring CXCL13 of patients with CVID. There was no statistically significant correlation identified between these two parameters (r: +0.06, p: 0.6, figure S6a).

**Figure S6**

a)

b)

c)

**Figure S6. a)** Correlation between the levels of CXCL13 in the sera of 76 patients with CVID and their age during venepuncture. **Left**, all patients with CVID; **middle/blue circles**, female patients, **right/red circles**, male patients. r: correlation coefficient; + indicates positive correlation for r between 0 and 1. eir age duringgative correlation for r between -1 and 0. As data did not follow Gaussian distribution, correlation was evaluated using Spearman analysis. ns indicates no statistical significance of the correlation (statistical significance for p<0.05)**. b)** The change of CXCL13 values in sera of CVID patients when their serum CRP values change from normal to high. Two repeated measurements of CXCL13 values in sera of 13 CVID patients and their corresponding CRP categories “normal” and “high”. “normal CRP” for CRP values of ≤5 mg/l and “high CPR” for values of >5mg/l. Each horizontal line joins the two repeated measurements of CXCL13 values i.e., one for “normal CRP” and one for “high CRP” in the same individual and represents each individual patient with CVID. Y axis was interrupted into 3 segments for visualisation purposes. The paired analysis was evaluated by Wilcoxon test (ns indicates no statistical significance for p<0.05).

**c)** CXCL13 levels in serum for 10 patients with CVID compared before and after the initiation of immunoglobulin G replacement. Each horizontal line joins the measurements for CXCL13 before and after the initiation of immunoglobulin replacement in the same patient. The paired analysis was performed by Wilcoxon test. ns indicates no statistical significance of the correlation (statistical significance for p<0.05)**.**

**No difference in the number of complications between female and male patients with CVID**

Due to the fact that in our cohort of patients with CVID females had higher levels of CXCL13 than males, we wanted to explore whether they have higher number of complications as well. Including (data not shown) or excluding bronchiectasis, more female patients had greater number of complications than their male counterparts, 1 female having 6 complications and 6 females having 5 complications as compared to 0 males and 1 male respectively (see figure S7). The average number of complications in female patients was higher than in male patients. However, the difference of their medians was not statistically significant (p=0.32). Interestingly, there was no statistical difference between the 2 sexes for the number of complications using contingency analysis and χ^2^ for trend (p=0.16, table S2, figure S7).

**Figure S7.** Number of CVID-related complications compared between male and female patients with CVID. Horizontal lines represent median number of complications. CVID: patients with CVID; n: number of individuals in each gender group. Data were statistically analysed by the Mann-Whitney non-parametric test. ns indicates no statistical significance of the comparison (statistical significance for p<0.05)**.**

| **Table S2.** Number of complications per gender of patients with CVID | | |
| --- | --- | --- |
| Number of complications | Female CVID (n=33) | Male CVID (n=26) |
| 0 complications | 8 | 7 |
| 1 complication | 8 | 8 |
| 2 complications | 6 | 5 |
| 3 complications | 2 | 4 |
| 4 complications | 2 | 1 |
| 5 complications | 6 | 1 |
| 6 complications | 1 | 0 |
| n: number of individuals;  χ^2^ for trend: statistical significance for *, p<0.05; **, p<0.01; ***, p<0.001; ****, p<0.0001 | | |

**Figure S8.** Correlation between CXCL13 values in sera of patients with CVID and their corresponding number of complications. Correlation between CXCL13 values in sera of 59 CVID patients and their corresponding number of complications. (Top) Correlation between CXCL13 values in sera of 32 females from the 59 CVID patients and their corresponding number of complications. (Bottom) Correlation between CXCL13 values in sera of 27 male from the 59 CVID patients and their corresponding number of complications. R: correlation coefficient; + indicates a positive correlation for R between 0 and 1. The correlation was evaluated using Spearman analysis. (*** indicate statistical significance of the correlation for p<0.001, **** statistical significance for p<0.0001)**.**

**Sensitivity Analysis and Collinearity Assessment Among CVID Complications in Relation to CXCL13 Levels**

**Figure S9.** Pairwise correlation matrix assessing collinearity for CVID complications as per their association to serum CXCL13 levels. Spearman’s analysis. r: correlation coefficient between -1 and 1.

| **Table S3.** The p-values from Pearson’s pairwise correlation between complications of CVID | | | | | | |
| --- | --- | --- | --- | --- | --- | --- |
| p-values | **splenomegaly** | **autoimmunity** | **enteropathy** | **lymphadenopathy** | **cytopenia** | **granulomata** |
| **splenomegaly** |  | 0.59 (0.90)* | 0.71 (1.00) | 0.96 (1.00) | 0.23 (0.86) | **0.05** (0.38) |
| **autoimmunity** | 0.59 (0.90) |  | 0.25 (0.75) | 0.51 (0.96) | 0.41 (0.88) | 0.64 (0.98) |
| **enteropathy** | 0.71 (1.00) | 0.25 (0.75) |  | **0.008** (0.12) | 0.31 (0.78) | 0.65 (0.98) |
| **lymphadenopathy** | 0.96 (1.00) | 0.51 (0.96) | **0.008** (0.12) |  | 0.55 (0.96) | 0.50 (0.94) |
| **cytopenia** | 0.23 (0.86) | 0.41 (0.88) | 0.31 (0.78) | 0.55 (0.96) |  | 0.18 (0.9) |
| **granulomata** | **0.05** (0.38) | 0.64 (0.98) | 0.65 (0.98) | 0.50 (0.94) | 0.18 (0.9) |  |
| Statistically significant p-values ≤ 0.05 are shown in **bold**. * indicates adjusted p-values in parentheses after Benjamini-Hochberg (FDR) multiple comparison adjustments. | | | | | | |

**Correlation between CXCL13 values in the sera of patients with CVID and their corresponding immune phenotype**

**Figure S10.** Correlation between CXCL13 values in the sera of patients with CVID and their corresponding immune phenotype. (Left) Correlation between CXCL13 values in sera of 39 CVID patients and their corresponding frequencies of CD21^low^ B cells. (Right) Correlation between CXCL13 values in sera of 39 CVID patients and their corresponding frequencies of switched memory B cells. R: correlation coefficient; + indicates positive correlation for R between 0 and 1. - indicates negative correlation for R between -1 and 0. The correlation was evaluated using Spearman analysis. ns indicates no statistical significance of the correlation (statistical significance for p<0.05)**.** smB- indicates low frequency of switched memory B cells.

**Frequency of peripheral cTfh cell subsets in CVID patients and healthy controls.**

We compared the patients with CVID and the healthy controls for their peripheral cTfh cell subsets frequency. Overall, patients with CVID had a higher frequency of total circulating Tfh cells (cTfh) compared with healthy controls (p=0.0002, table S4).

In terms of cTfh cell subsets, patients with CVID have a higher frequency of cTfh1 (p<0.0001) and a lower frequency of cTfh2 (p=0.0018) and cTfh17 (p=0.0014) compared with healthy controls.

Interestingly, patients with CVID have a higher frequency for all cTfh cell subsets expressing both PD1 and ICOS or only PD1, compared with healthy controls: PD1+ICOS+ cTfh1 (p<0.0001), PD1+ICOS+cTfh2 (p<0.0001), PD1+ICOS+cTfh17 (p=0.0034), PD1+ICOS-cTfh1 (p=0.003), PD1+ICOS- cTfh2 (p<0.0001) and PD1+ICOS- cTfh17 (p=0.0159).

| **Table S4**. Comparison between CVID patients and HD for the frequencies of  cTfh cell subsets | | | | | | |
| --- | --- | --- | --- | --- | --- | --- |
| **cTfh subsets** | **CVID (vs. HD)** | **CVID**  (%) | **HD**  (%) | **p - value** | **Adj. p-value**  **(FDR)** | |
| cTfh | **↑^a^** | 12.64 | 09.05 | 0.0002  *** | 0.0004  *** | |
| cTfh1 | **↑** | 29.90 | 23.86 | <0.0001  **** | 0.00025  *** | |
| cTfh2 | **↓^b^** | 34.00 | 38.04 | 0.002  ** | 0.00286  ** | |
| cTfh17 | **↓** | 24.56 | 28.20 | 0.001  ** | 0.00167  * | |
| PD1+ICOS+ cTfh1 | **↑** | 13.55 | 08.44 | <0.0001  **** | 0.00025  *** | |
| PD1+ICOS+ cTfh2 | **↑** | 05.90 | 03.58 | <0.0001  **** | 0.00025  *** | |
| PD1+ICOS+ cTfh17 | **↑** | 06.53 | 04.28 | 0.003  ** | 0.00333  ** | |
| PD1+ICOS- cTfh1 | **↑** | 32.43 | 27.39 | 0.003  ** | 0.00333  ** | |
| PD1+ICOS- cTfh2 | **↑** | 18.50 | 11.47 | <0.0001  **** | 0.00025  *** | |
| PD1+ICOS- cTfh17 | **↑** | 13.30 | 11.08 | 0.02  * | 0.02000  * | |
| cTfh subset frequencies (median %) from venous blood of CVID patients and HD were analysed with flow cytometry (see material and methods). Arrow symbol pointing upwards^a^ indicates higher frequencies as compared to the arrow symbol pointing downwards^b^ indicating lower frequencies. As data were not distributed normally, statistical significance was tested with the Mann-Whitney non-parametric analysis (p<0.05). To correct for multiple comparisons across 10 subset analyses, the Benjamini-Hochberg procedure was applied to control the false discovery rate (FDR). All comparisons remained statistically significant following FDR correction (*adjusted p* < 0.05). * for p<0.05, ** for p<0.01, *** for p<0.001, **** for p<0.0001. | | | | | |  |

**Figure S11. Representative gating strategy and frequencies of peripheral cTfh cell subsets from PBMCs of a a) healthy donor [left panel] and b) CVID patient [right panel].** Gating frequencies are shown below each plot.

**Serum levels of CXCL13 in patients with XLA**

We wanted to explore what were the serum levels of CXCL13 in 7 patients with X-linked agammaglobulinemia a hereditary primary immunodeficiency syndrome^2^. In this disease, there are less than 2% CD19^+^B cells, marked hypogammaglobulinemia, decreased Tfh cells in the circulation, and lack of germinal centre formation^42,55,68^. We observed that the median of CXCL13 values in XLA (48.57 pg/ml) was similar to the median in HD (p=0.11) and much lower than the median of the CVID patients (p<0.0001, figure S12)

**Figure S12.** Comparison between serum CXCL13 levels in CVID vs HD and vs XLA. Horizontal lines represent median values of CXCL13, error bars represent 75% and 25% percentile. Y axis was interrupted into 2 segments for visualisation purposes. CVID: Common Variable Immunodeficiency; HD: healthy donors; XLA: X-linked agammaglobulinemia; n: number of individuals tested. Data were statistically analysed by the Mann-Whitney non-parametric test (**** statistical significance for p<0.0001, * statistical significance for p<0.05).
